# The Multichannel Intraoesophageal Impedance Transit Concept

**DOI:** 10.7759/cureus.73396

**Published:** 2024-11-10

**Authors:** Ismail Miah, Terry Wong, Sebastian Zeki, Jafar Jafari

**Affiliations:** 1 Faculty of Life Sciences and Medicine, King's College London, London, GBR; 2 Gastroenterology, Guy's and St Thomas' NHS Foundation Trust, London, GBR

**Keywords:** achalasia, barium, impedance, oesophagus, transit

## Abstract

Introduction/aims

The multichannel intraoesophageal impedance transit (MIIT) is a new clinical concept that is being introduced to measure the oesophageal transit during a 24-hour multichannel impedance-pH (MII-pH) study.

Methods

MIIT was tested in a case-control study between January 2020 and December 2023. A laboratory test was first conducted to determine the saline baseline impedance (SBI) using MII-pH catheters. SBI was later used to reference the oesophageal transit of saline during the MIIT test. In the MIIT test, patients rapidly drank 200 ml of saline within 20 seconds. The saline transit was identified by the temporal impedance changes from the oesophageal mucosal baseline impedance (MBI) to the approximate SBI level. The duration of SBI was recorded from multiple impedance sensors in the proximal, mid and distal oesophagus which form the MIIT. The regional MIIT were correlated to the Hospital Odynophagia Dysphagia Questionnaire (HODQ) scores for clinical dysphagia and severity. Regional MIIT were also compared between achalasia patients (case group) and non-achalasia patients (control group) based on high-resolution manometry (HRM) and barium swallow (BS) diagnosis. Descriptive statistics, *t*-test and chi-squared test compared the MIIT with respect to HODQ scores, HRM and BS outcomes. Receiver operating characteristic curves with Youden's J indices determined the optimal MIIT cut-off threshold for achalasia.

This research was approved by the North West Haydock NHS Health Research Authority (REC 18/NW/0120) and the Integrated Research Application System (Project ID: 333800).

Results

Nine hundred and eleven patients undertook the MIIT test prospectively to HRM study (females 554, mean age 50.9 years). Three hundred and thirty-three patients (36.6%) additionally underwent the BS study (the BS-HRM diagnostic concordance outcome was 83.8%). Oesophageal luminal transit of saline was identifiable during MIIT and found to be significantly lower than the MBI (*t*-value=3.59-9.07, p<0.001). Regional MIIT increased with higher dysphagia severity (r≈0.33, p<0.001) and positive HODQ scores for clinical dysphagia (*t*-value=6.18-6.30, p<0.001). Similarly, prolonged regional MIIT was observed in achalasia patients based on BS study diagnosis (*t*-values: 9.86-11.2, p<0.001) and HRM study diagnosis (*t*-values: 23-27.4, p<0.001). Patients with concordant BS-HRM study for achalasia also showed prolonged regional MIIT (*t*-value: 13.9-16.4, p<0.001). The optimal MIIT of the distal oesophagus for achalasia diagnosis is between 4.05 minutes and 5.45 minutes (sensitivity: 73.8-100%, positive predictive values: 90.5-94.4%). MIIT thresholds for achalasia show higher concordance to the HRM study than the BS study at 4.05 minutes (χ^2^=4.69, p<0.030).

Conclusions

The MIIT concept was demonstrated to be a simple and effective transit assessment that showed exceptional reliability to BS and HRM studies. The MIIT technique can be easily incorporated into the MII-pH investigation without causing additional risk or burden to patients.

## Introduction

Dysphagia symptoms may be troublesome for some patients and their clinicians, which can pose risks of malnutrition, difficulty ingesting medication and aspiration pneumonia. Patients may feel debilitated by their dysphagia and odynophagia symptoms which can be associated with weight loss, reduction in quality of life or impairment in lifestyle. This may compel patients to seek treatment. In cases of extreme dysphagia symptoms, clinical management may entail prolonged hospital admission or supporting them in intensive care. The dysphagia symptoms may occur from benign oesophageal disorders that can result from impaired lower oesophageal sphincter (LOS) relaxation pathology and/or severe oesophageal body dysmotility. Ultimately, either condition may contribute to the poor oesophageal bolus transit that causes patients dysphagia symptoms. Repetitive and prolonged intraoesophageal bolus retention can lead to developing anatomical abnormalities, such as dilated oesophagus, diverticulum and megaoesophagus, which increase the risks of oesophageal perforation. Thus, early detection of poor oesophageal transit permits clinicians to implement measures to prevent sinister anatomical abnormalities from occurring.

The guideline for oesophageal transit monitoring has recently been incorporated in the latest edition of the Chicago Classification version 4.0 (CCv4.0) [1]. CCv4.0 recommends patients be referred for a barium swallow (BS) study to confirm suspected major motility disorders found on high-resolution manometry (HRM), secondly when HRM findings are equivocal or the dysphagia symptoms in patients could not be explained by the HRM [1]. In clinical practice, oesophageal physiology investigation routinely assesses the oesophageal motor function and gastric reflux and not the oesophageal transit (which is currently an unmet clinical need to measure transit in oesophageal physiology). Abnormality in the oesophageal transit is a common cause of the dysphagia symptoms, and the oesophageal dysmotility measured in clinical physiology can explain the reason for poor oesophageal transit. Concurrently, both transit and motility are complementary to each other and could be investigated in parallel from a single clinical test if the concept proposed in this paper is fruitful. Both motility and transit would provide better insights into the pathophysiology of the dysphagia/odynophagia symptoms and the selection of the most appropriate treatment to alleviate the symptoms. In the current clinical practice, the oesophageal transit assessment requires additional clinical referral to radiology and a separate hospital appointment for the patient to undergo a BS study with X-ray imaging. The administrative process and waiting time for the oesophageal transit test on BS are likely to delay the patient's diagnosis, and subsequently, their treatment is delayed.

Oesophageal transit testing has been attempted during HRM combined with multichannel impedance (HRMZ) studies [2-7]. However, the HRMZ technology is relatively new for measuring oesophageal transit and has demonstrated pitfalls (i) from the restrictive timeframe in performing HRMZ study (HRMZ study is a stationary test and would not be suitable for measuring prolonged transit time in patients with major motility disorders such as achalasia), (ii) the interpretation of oesophageal bolus retention on the HRMZ study is subjectively based on a purple colour scale gauge and (iii) HRMZ recording being switched to the conventional line plot mode shows incoming manometry waveforms obscuring the impedance trace that measures the oesophageal transit [6].

This article addresses the potential use of an alternative device that would exclude the pitfalls associated with the HRMZ study for oesophageal transit investigation. The proposed concept is to utilise the 24-hour multichannel impedance-pH (MII-pH) catheter to measure oesophageal transit from the impedance changes (MII-pH study is a well-known clinical test for objectively measuring gastroesophageal reflux disease). The concept being introduced to measure the oesophageal transit during the MII-pH study is referred to as the multichannel intraoesophageal impedance transit (MIIT) test.

## Materials and methods

This research project was hosted by Guy's and St Thomas' NHS Foundation Trust in affiliation with King's College London (University of London) in the United Kingdom. Patients referred to the Oesophageal Laboratory for the MII-pH study, as part of their standard clinical care between January 2020 and December 2023, were invited to participate in this project and undertake the MIIT test. The enrolment process involved obtaining patients' consent for measuring oesophageal transit during their MII-pH study. Written consents were obtained from participants to use their clinical data for teaching, research and service audits. The protocols of this research fulfilled the Declaration of Helsinki and were approved by the North West Haydock NHS Health Research Authority (REC 18/NW/0120) and the Integrated Research Application System (Project ID: 333800).

Patient selection

The MIIT concept was tested in a case-control study in adult patients with achalasia (case group) and non-achalasia (control group). Selecting patients was strict to those clinically referred for MII-pH studies as part of their standard clinical care. The MII-pH study was performed as per routine practice with the MIIT test being conducted as a complementary test. The MII-pH study was performed prospectively to the BS and HRM investigations. The HRM study diagnosis for achalasia was based on CCv4.0, while the BS study diagnosis for achalasia was based on radiological features of contrast retention or delayed clearance along with at least one anatomical abnormality feature (distal oesophageal dilatation, narrowing oesophagogastric junction (OGJ), "bird's beak" or "rat's tail" appearance on X-ray imaging). The absence of these radiological makers would exclude achalasia from the BS study. A validated Hospital Odynophagia Dysphagia Questionnaire (HODQ) form was also filled out by patients as part of their diagnostic evaluation for gauging the dysphagia severity [8].

MII-pH device specification

The MII-pH catheters being used for the MIIT test were ComforTEC Z/pH probe brand (reference ZAI-BG-44, manufactured by Diversatek Healthcare, Highlands Ranch, Colorado, United States). These catheters were single-use items per patient and have the physical dimensions of 2.3 mm in diameter and 70 cm in length. The catheters have polyvinyl covering with protruding two pH sensors that are 10 cm apart and have six longitudinally paired impedance sensors that provide oesophageal body impedance coverage up to 18 cm above manometric OGJ. The MII-pH catheters were calibrated following the manufacturer's guidelines in pH buffer solutions 4 and 7. The recording monitor used for the MII-pH study was a ZepHr® Impedance/pH Reflux Monitor (manufactured by Diversatek Healthcare, Highlands Ranch, Colorado, United States).

Saline baseline impedance (SBI)

**Figure 1:**
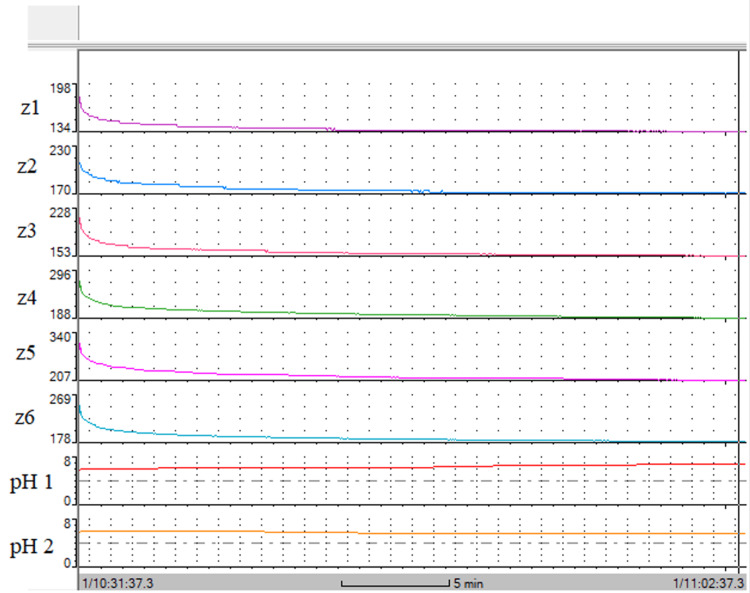
The approximated nadir SBI during the laboratory testing. The impedance sensors (z1, z2, z3, z4, z5 and z6) were submerged in saline for approximately 30 minutes. At the onset of submerging, the impedance sensors' initial readings were between 198 Ω and 340 Ω. Impedance stabilisation occurred approximately 10 minutes after soaking the impedance in saline. SBI: saline baseline impedance

SBI was measured from a small number of MII-pH catheters after the calibration phase. This was simply performed by inserting the MII-pH catheters into a vertically upright test tube (25 cm) that contained the saline (0.9% w/v NaCl concentration). All the pH and impedance sensors were submerged in the saline at room temperature for 30 minutes. The BioView Analysis software (version 5.7.1.0) (Sandhill Scientific Inc., Diversatek Healthcare, Highlands Ranch, Colorado, United States) was used to analyse the in vitro laboratory test recording of saline and to approximate the SBI (see Figure 1). The SBI reference was considered from the plateau region of the recording (this would be the nadir impedance of SBI). This SBI reference was used in MIIT studies in patients to identify the saline transit in the oesophagus.

Patient preparation

The patients prepared for the MII-pH test by stopping their anti-reflux medication (proton pump inhibitors for five days, H2 antagonist and prokinetic therapy for two days and anti-acids for 24 hours). Patients taking antiplatelet agents or undergoing anticoagulant therapy were requested to discontinue the treatment temporarily for two days before testing, and international normalised ratio tests were performed for the safety of clinical testing (i.e. less than 3 was deemed safe). Patients were fasting from solid food for six hours and were nil by mouth for two hours before testing.

Intubation and positioning of the MII-pH catheters

After the consultation and consenting process, patients were sprayed with xylocaine (lidocaine 30 mg/spray) between three and five times in the nasopharynx using the nozzle provided (Aspen Pharma Trading Limited, Dublin 24, Ireland). The distal portion of the MII-pH catheter was smeared with lubrication gel (5 cm from the tip), and the catheter was inserted into the anaesthetised nostril to assess for patient toleration and resistance within the nasal cavity. If the nasal intubation was successful, patients were asked to drink water, and the catheter was slowly advanced into the cricopharyngeus and then into the oesophagus. The intubation was continued into the stomach by additional swallowing manoeuvres until the catheter was inserted approximately 50 cm from the nose and the distal pH sensor detected an acidic pH reading of the stomach (pH <4). The MII-pH catheter was then re-adjusted to position the oesophageal pH sensor to 5 cm above the proximal manometric LOS which registered neutral oesophageal pH [9]. The distal pH sensor was automatically positioned 5 cm below the manometric LOS that continued to measure the gastric acidic pH. In this arrangement of the pH sensors and based on the catheter design, the array of six paired impedance sensors was distributed along the oesophageal body at 3 cm (z6), 5 cm (z5), 7 cm (z4), 9 cm (z3), 15 cm (z2) and 17 cm (z1) above the manometric LOS. The placement of the oesophageal pH sensor allowed the detection of gastric reflux events (if they were to occur during the MIIT test). Any acid reflux events occurring during the MIIT test were excluded from this study.

To distinguish between the SBI and the oesophageal mucosal baseline impedance (MBI) during the MIIT study, the simple method to calculate the mean nocturnal baseline impedance (MNBI) during the MII-pH study was adopted [10]. This was performed on a cohort of patients with concordant HRM and BS study findings for either achalasia (case group) or exclusion of achalasia (control group). The MBI found in patient groups were compared to the laboratory findings of SBI.

MIIT test protocol

Patients were seated in the upright position with the MII-pH catheter being appropriately positioned in the oesophagus (see section "Intubation and positioning of the MII-pH catheters"). Patients were requested to inhale and rapidly drink 200 ml of saline through a straw (or as much as possible) from a cup within 20 seconds. The rapid drinking of 200 ml of saline was adopted from the protocol to measure the oesophageal transit on HRMZ testing [7]. The drinking phase of saline was recorded as the first meal event on the reflux monitor, which was performed within the first 10 minutes of the MII-pH study recording. Patients remained in their seated position for a further 15 minutes to receive instructions on how to use the reflux monitor and record their symptoms, mealtimes and body positional changes. They were also advised to conduct their normal routine day during the MII-pH study and comply with certain dietary and lifestyle restrictions (i.e. avoiding acidic food and drink, not drinking alcoholic beverages, not getting the reflux monitor wet, etc.).

**Figure 2:**
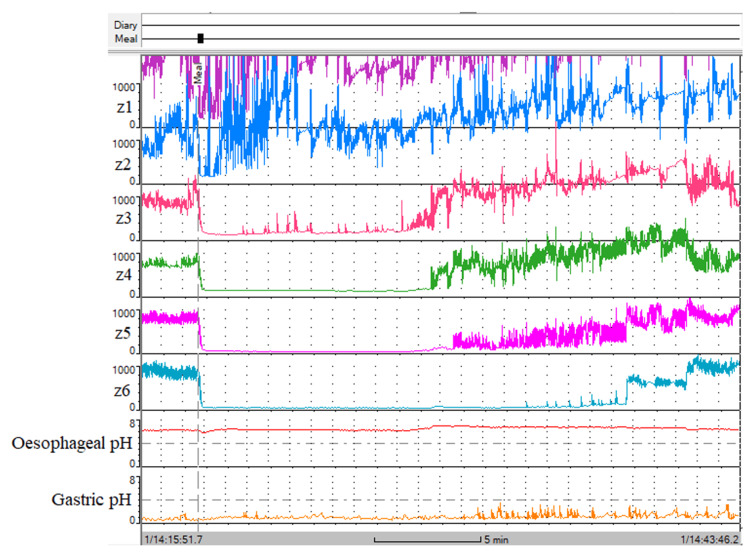
The MIIT test in a patient diagnosed with achalasia on HRM and BS studies. The MIIT recording is being viewed in a window frame that is 26 minutes in length. Notably, the MIIT study presented in an antegrade fashion with MBI identified prior to saline drinking and again after the oesophageal clearance of saline. In between the observation of the MBI, the SBI approximation is observed from the saline retention at z1 (not measurable from significant noise), z2 (1.10 minutes), z3 (10.9 minutes), z4 (11.8 minutes), z5 (19.9 minutes) and z6 (22.7 minutes). MIIT: multichannel intraoesophageal impedance transit; HRM: high-resolution manometry; BS: barium swallow; MBI: mucosal baseline impedance; SBI: saline baseline impedance

The BioView Analysis software (version 5.7.1.0) was used to view and analyse the MII-pH recording including the MIIT test. The MIIT time of saline was investigated in the proximal, mid and distal oesophagus which corresponded to the saline transit measurements captured on impedance sensors z2 (located 15 cm above the LOS), z3 (located 9 cm above the LOS) and z6 (located 3 cm above the LOS) (see Figure 2). At each oesophageal location, MIIT time was calculated based on the duration of the impedance drop to the approximated SBI level (this occurred when the saline was detected in the oesophageal lumen). As the oesophagus discharges the saline into the stomach, there is an impedance recovery to the MBI [2,3,11]. The SBI detected within the oesophageal lumen during the MIIT test was considered from a close approximation of the SBI found in the laboratory tests. The SBI during the MIIT is taken to be lower than the MBI (the MBI was observed prior to drinking the saline and again after the oesophageal clearance of saline). The duration of the approximated SBI during the MIIT was considered as the saline retention period.

Statistical method

The statistical analysis included computing for the descriptive data of SBI, MBI and MIIT (mean, 5-95% confidence interval, median, 25-75% quartile ranges and Pearson correlation). Data comparison was appropriately performed using t-test, chi-squared (χ2) and analysis of covariance. This was to assess for statistical significance, and a p-value of <0.05 was considered to be statistically significant.

Comparing MIIT in Oesophageal Regions

MIIT findings in the proximal, mid and distal oesophagus were correlated to dysphagia severity based on HODQ scores. The regional MIIT were also compared between normal and positive HODQ scores for clinical dysphagia (scores >6.4 were considered positive) [8]. Finally, regional MIIT were compared between patients with achalasia (case group) and non-achalasia (control group) based on BS studies and HRM studies and when both BS and HRM studies revealed concordant findings.

MIIT Cut-Off Thresholds for Achalasia Diagnosis

Receiver operating characteristic (ROC) curves with maximum Youden's J indices were used to determine the optimal MIIT cut-off thresholds of the distal oesophagus to diagnose achalasia. This was computed with respect to HRM studies and BS studies and for concordant findings of both BS and HRM tests for achalasia. The MIIT cut-off thresholds for achalasia were further quantified for the positive predictive value (PPV), negative predictive value (NPV), odds ratio (OR) and likelihood ratio (LR+) for achalasia to be found. The C-statistics of the ROC curve model was calculated to evaluate each model.

MIIT Cut-Off Threshold Concordance for Clinical Dysphagia and Achalasia

MIIT thresholds found for clinical dysphagia (based on HODQ scores) and achalasia were prospectively assessed for concordance to BS and HRM studies.

## Results

Patient demographics

The MIIT test was performed on 911 patients (female/male=554:357, mean age 50.9 years (range=18-88 years)). All patients had an HRM study before the MII-pH study and the MIIT test. HRM revealed achalasia in 97 of the 911 patients (10.6%). In addition, 333 patients of the 911 (36.6%) had a BS study, and achalasia was found in 106 patients (31.8%). BS studies statistically revealed higher achalasia diagnostic prevalence compared to HRM (χ2=80.1, p<0.001). Two hundred and seventy-nine of the 333 patients (83.8%) showed BS studies concordant to HRM in diagnosing achalasia or excluding achalasia.

The rapid drinking of saline (200 ml) within 20 seconds was successfully performed by all the patients from the control group (i.e. achalasia was excluded by either the HRM or BS study). In the case group, approximately 20% of patients had difficulty drinking the full volume of saline, and the remaining volume of saline found was ≤50 ml. None of the patients in the case group or the control group reported the saline being vomited.

Laboratory SBI and oesophageal MBI comparison

The SBI found in the laboratory test and the MBI found in patients are outlined in Table 1. The SBI was found to be significantly lower compared to the MBI found in patients from the control group (174 Ω vs. 2025 Ω, t-value=9.07, p<0.001) and from the case group (174 Ω vs. 856 Ω, t-value=3.59, p<0.001). Incidentally, the MBI in patients with achalasia (case group) was statistically significantly lower compared to the MBI found in non-achalasia patients (control group) (t-value=8.6, p<0.001). The choice of transit marker could impact the MIIT analysis such that a transit marker with baseline impedance >600 Ω in the laboratory testing would not be useful for the MIIT test in suspected achalasia. This is because MBI in achalasia patients was found to be as low as 640 Ω (5% CI) and oesophageal transit of substance with similar SBI would not be observed on MIIT. However, the SBI being significantly lower than the MBI in patients (from both control and case groups) in this study permitted distinguishing the oesophageal luminal saline transit from MBI during the MIIT test. The saline transit was identifiable at various impedance settings (from 0-500 Ω and up to 4000 Ω). In the laboratory test, the nadir impedance of saline (or SBI) was observed after 10 minutes of submerging the impedance sensors. The initial SBI registered is as high as ~300 Ω in two of the five impedance sensors. This level of SBI was observed in fast or rapid oesophageal clearance of saline particularly in the control group.

**Table 1:** The SBI found in the laboratory test and the MBI found in patients who had concordant HRM and BS studies for achalasia (case group) and non-achalasia (control group). The SBI values recorded were observed at the stabilised phase which was approximately after 10 minutes of soaking the impedance sensors in saline. The MBI in 18 patients were excluded from this table owing to patients not indicating their recumbent period during the MII-pH study or the MII-pH study was incomplete of the nocturnal recording. SBI: saline baseline impedance; MBI: mucosal baseline impedance; HRM: high-resolution manometry; BS: barium swallow; MII-pH: multichannel impedance-pH

Baseline impedance (Ω)	N	Mean (5-95% CI)	Median (quartiles)
SBI	25	174 (156.3-191.8)	172.8 (140.7-207.1)
MBI (control group)	197	2025 (1891-2159)	2027 (1306-2598)
MBI (case group)	64	856.4 (634.6-1078)	487 (200-1013)

MIIT and dysphagia symptoms based on HODQ scores

**Figure 3:**
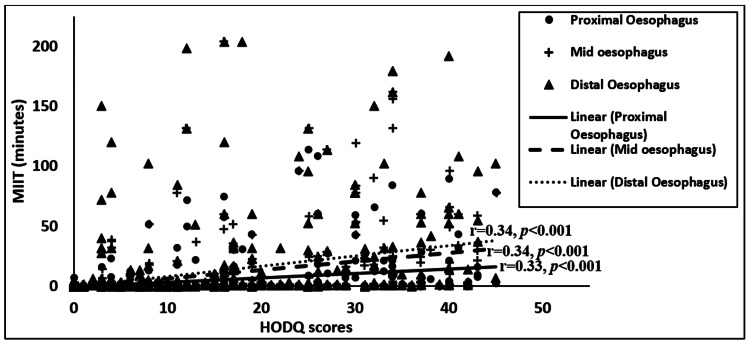
The MIIT correlation to dysphagia severity (based on HODQ scores) in the proximal, mid and distal oesophagus. MIIT: multichannel intraoesophageal impedance transit; HODQ: Hospital Odynophagia Dysphagia Questionnaire

Six hundred and ninety-nine patients completed the HODQ form, and the Pearson correlation from this dataset revealed trends in higher dysphagia severity (based on HODQ scores) correlating to increased MIIT time in the proximal oesophagus (slope 0.36, r=0.33, p<0.001), mid oesophagus (slope 0.71, r=0.34, p<0.001) and distal oesophagus (slope 0.85, r=0.34, p<0.001) (see Figure 3). The differences in slopes were not statistically significant to suggest the dysphagia pertains from one regional transit rate to another (F=0.75, p=0.47).

Of the 699 patients, 334 patients (47.8%) had clinical dysphagia based on positive HODQ scores and also demonstrated significantly prolonged MIIT in the proximal oesophagus (6.1 minutes vs. 0.53 minutes, t-value=6.18, p<0.001), mid oesophagus (11.6 minutes vs. 0.90 minutes, t-value=6.22, p<0.001) and distal oesophagus (14.5 minutes vs. 2.2 minutes, t-value=6.30, p<0.001) (see Table 2). The MIIT at the 95% CI for patients without clinical dysphagia were found to be <0.71 minutes in the proximal oesophagus, <1.29 minutes in the mid oesophagus and <3.29 minutes in the distal oesophagus. Eighty-four of the 175 patients (48%) without achalasia based on concordant HRM and BS study findings had normal HODQ scores, whereas three of 47 patients (6.38%) with concordant achalasia findings on both standard clinical tests have normal HODQ scores (χ2=26.9, p<0.001).

**Table 2:** MIIT times of saline (in minutes) found in the proximal, mid and distal oesophagus with respect to HODQ scores (normal scores vs. positive scores for clinical dysphagia). MIIT: multichannel intraoesophageal impedance transit; HODQ: Hospital Odynophagia Dysphagia Questionnaire

	Normal HODQ scores (n=365)		Positive HODQ scores (n=334)			
Oesophageal region	Mean (5-95% CI)	Median (quartiles)	Mean (5-95% CI)	Median (quartiles)	t-value	p-value
Proximal	0.53 (0.35-0.71)	0.24 (0.16-0.39)	6.1 (4.3-8.0)	0.31 (0.19-0.76)	6.2	<0.001
Mid	0.90 (0.52-1.30)	0.31 (0.19-0.51)	11.6 (8.1-15.1)	0.47 (0.25-1.70)	6.2	<0.001
Distal	2.20 (1.1-3.29)	0.46 (0.28-0.97)	14.5 (10.6-18.4)	0.79 (0.40-3.20)	6.3	<0.001

MIIT correlation to BS study

The 106 patients (31.8%) with positive BS study for achalasia showed significantly prolonged MIIT in the proximal oesophagus (18.8 minutes vs. 0.73 minutes, t-value=9.9, p<0.001), mid oesophagus (36.9 minutes vs. 1.25 minutes, t-value=10.1, p<0.001) and distal oesophagus (45 minutes vs. 2.40 minutes, t-value=11.2, p<0.001) (see Table 3 for details). The ROC curve at the maximum J-index revealed the optimal MIIT cut-off threshold in the distal oesophagus to diagnose achalasia with respect to the BS outcome to be 5.45 minutes (sensitivity 73.8% and specificity 96.5%, χ2=184.3, p<0.001) (see Table 4). This cut-off threshold produced a PPV of 90.5% and an NPV of 89%. The LR+ and OR were 21 and 77.1, respectively.

**Table 3:** The MIIT times of saline (in minutes) found in the proximal, mid and distal oesophagus with respect to the BS study outcome. MIIT: multichannel intraoesophageal impedance transit; BS: barium swallow

	Control group (n=227)		Case group (n=106)			
Oesophageal region	Mean (5-95% CI)	Median (quartiles)	Mean (5-95% CI)	Median (quartiles)	t-value	p-value
Proximal	0.73 (0.21-1.25)	0.23 (0.16-0.35)	18.8 (13.4-24.2)	7.7 (0.51-22.0)	9.86	<0.001
Mid	1.25 (0.51-2.00)	0.28 (0.19-0.51)	36.9 (26.5-47.2)	18.2 (1.20-54.5)	10.1	<0.001
Distal	2.36 (0.82-3.90)	0.50 (0.26-0.89)	45.0 (34.3-55.7)	30.0 (4.0-60.0)	11.2	<0.001

**Table 4:** The ROC curve analysis of MIIT of the distal oesophagus for achalasia with respect to BS outcome (AUC 0.885, 95% CI (0.839-0.931)). MIIT: multichannel intraoesophageal impedance transit; PPV: positive predictive value; NPV: negative predictive value; LR+: likelihood ratio; OR: odds ratio; ROC: receiver operating characteristic; BS: barium swallow; AUC: area under curve

MIIT (minutes)	Sensitivity (%) (5-95% CI)	Specificity (%) (5-95% CI)	PPV	NPV	LR+	OR
>4.70	73.8 (64.2-82.0)	96.0 (92.1-97.9)	88.4	89.0	16.8	61.1
>5.10	73.8 (64.2-82.0)	96.0 (92.6-98.2)	89.4	89.0	18.6	68.2
>5.45	73.8 (64.2-82.0)	96.5 (93.2-98.5)	90.5	89.0	21.0	77.1
>5.70	72.8 (63.2-81.1)	96.5 (93.2-98.5)	90.4	88.7	20.7	73.3
>6.10	71.8 (62.1-80.3)	96.5 (93.2-98.5)	90.2	88.3	20.4	69.9

MIIT correlation to HRM study

The 97 patients with positive achalasia diagnosis on HRM (10.6%) demonstrated significantly prolonged MIIT in the proximal oesophagus (25.5 minutes vs. 0.57 minutes, t-value=23.0, p<0.001), mid oesophagus (48.1 minutes vs. 1.01 minutes, t-value=23.3, p<0.001) and distal oesophagus (60.9 minutes vs. 1.8 minutes, t-value=27.4, p<0.001) (see Table 5). The ROC curve at the maximum J-index revealed the optimal MIIT cut-off threshold in the distal oesophagus to diagnose achalasia with respect to the HRM study to be 5.25 minutes (sensitivity 98.9% and specificity 97.3%, χ2=702.3, p<0.001) (see Table 6). The incidence of achalasia when MIIT is 5.25 minutes produced a PPV of 80.9% and an NPV of 99.9%. The LR+ and OR for achalasia were 36.4 and 3331, respectively.

**Table 5:** The MIIT times of saline (in minutes) found in the proximal, mid and distal oesophagus with respect to the HRM study outcome. MIIT: multichannel intraoesophageal impedance transit; HRM: high-resolution manometry

	Control group (n=814)		Case group (n=97)			
Oesophageal region	Mean (5-95% CI)	Median (quartiles)	Mean (5-95% CI)	Median (quartiles)	t-value	p-value
Proximal	0.57 (0.31-0.82)	0.26 (0.17-0.41)	25.5 (19.7-31.2)	16.1 (7.7-31.6)	23.0	<0.001
Mid	1.01 (0.46-1.56)	0.35 (0.20-0.62)	48.1 (37.6-58.6)	31.9 (18.2-60.0)	23.3	<0.001
Distal	1.75 (1.05-2.45)	0.58 (0.32-1.30)	60.9 (49.9-71.9)	40.0 (24.4-78.0)	27.4	<0.001

**Table 6:** The ROC curve analysis of MIIT of the distal oesophagus for achalasia with respect to HRM outcome (AUC 0.995, 95% CI (0.990-0.999)). MIIT: multichannel intraoesophageal impedance transit; PPV: positive predictive value; NPV: negative predictive value; LR+: likelihood ratio; OR: odds ratio; ROC: receiver operating characteristic; HRM: high-resolution manometry; AUC: area under curve

MIIT (minutes)	Sensitivity (%) (5-95% CI)	Specificity (%) (5-95% CI)	PPV	NPV	LR+	OR
>4.95	98.9 (94.2-100)	96.9 (95.5-98.0)	78.8	99.9	32.1	2920
>5.10	98.9 (94.2-100)	97.0 (95.6-98.1)	79.5	99.9	33.4	3045
>5.25	98.9 (94.2-100)	97.3 (95.9-98.3)	80.9	99.9	36.4	3331
>5.45	97.9 (94.2-99.7)	97.3 (95.9-98.3)	80.7	99.7	36.0	1647
>5.65	97.9 (92.5-99.7)	97.4 (96.1-98.4)	81.4	99.7	37.8	1728

MIIT correlation to concordant BS and HRM studies

The 70 patients of the 279 (25.1%) showing concordant findings for achalasia on BS and HRM studies revealed prolonged MIIT in the proximal oesophagus (27.9 minutes vs. 0.30 minutes, t-value=13.9, p<0.001), mid oesophagus (53.4 minutes vs. 0.45 minutes, t-value=13.6, p<0.001) and distal oesophagus (62.3 minutes vs. 0.81 minutes, t-value=16.4, p<0.001) (see Table 7). The ROC curve at the maximum J-index revealed the optimal MIIT cut-off threshold in the distal oesophagus for achalasia with respect to the concordant finding on BS and HRM study findings to be 4.05 minutes (sensitivity 100% and specificity 98.1%, χ2=251.2, p<0.001) (see Table 8). The incidence of achalasia when MIIT is 4.05 minutes produced a PPV of 94.4% and an NPV of 100%. The LR+ for achalasia to be found for MIIT being greater than 4.05 minutes was 52.3.

**Table 7:** The MIIT times of saline (in minutes) found in the proximal, mid and distal oesophagus with respect to concordant findings of the BS study and HRM study outcomes. MIIT: multichannel intraoesophageal impedance transit; HRM: high-resolution manometry; BS: barium swallow

	Control group (n=209)		Case group (n=70)			
Oesophageal region	Mean (5-95% CI)	Median (quartiles)	Mean (5-95% CI)	Median (quartiles)	t-value	p-value
Proximal	0.30 (0.25-0.34)	0.23 (0.16-0.34)	27.9 (20.6-35.3)	16.2 (8.0-41.3)	13.9	<0.001
Mid	0.45 (0.36-0.54)	0.28 (0.18-0.51)	53.4 (39.5-67.3)	36.2 (19.4-66.0)	13.6	<0.001
Distal	0.81 (0.64-0.98)	0.47 (0.28-0.87)	62.3 (49.1-75.5)	52.0 (28.4-78.0)	16.4	<0.001

**Table 8:** The ROC curve analysis of MIIT of the distal oesophagus for achalasia with respect to concordant findings of BS and HRM outcome (AUC 0.999, 95% CI (0.998-1.000)). MIIT: multichannel intraoesophageal impedance transit; PPV: positive predictive value; NPV: negative predictive value; LR+: likelihood ratio; OR: odds ratio; ROC: receiver operating characteristic; BS: barium swallow; HRM: high-resolution manometry; AUC: area under curve

MIIT (minutes)	Sensitivity (%) (5-95% CI)	Specificity (%) (5-95% CI)	PPV	NPV	LR+	OR
>3.25	100 (94.6-100)	97.1 (93.9-98.9)	91.8	100	34.8	-
>3.60	100 (94.6-100)	97.6 (94.5-99.2)	93.1	100	41.8	-
>4.05	100 (94.6-100)	98.1 (95.2-99.5)	94.4	100	52.3	-
>4.55	98.5 (92.0-99.9)	98.1 (95.2-99.5)	94.3	99.5	51.5	3382
>5.65	98.5 (92.0-99.9)	98.6 (95.9-99.7)	95.7	99.5	68.6	4532

MIIT concordance to clinical dysphagia and BS and HRM outcome

Data for this is outlined in Table 9. Based on HODQ scores, the prevalence of clinical dysphagia with MIIT exceeding 3.29 minutes in the distal oesophagus was found in 92 patients (27.5%). At this MIIT threshold for clinical dysphagia, we observed HRM to have statistically higher concordance to achalasia than found in BS studies (99% vs. 73.6%, χ2=11.29, p<0.001). Our proposed MIIT cut-off threshold for achalasia was at a higher MIIT duration from 4.05 minutes in the distal oesophagus which also revealed HRM to have statistically higher concordance to achalasia (99% vs. 72.6%, χ2=4.69, p=0.030). At higher MIIT thresholds of 5.25 minutes and 5.45 minutes, we did not observe a statistical difference in the concordance rates (MIIT at 5.25 minutes is 97.9% vs. 71.7%, χ2=2.56, p=0.110, and MIIT at 5.45 minutes is 96.9% vs. 71.6%, χ2=3.41, p=0.065).

**Table 9:** The MIIT cut-off thresholds and concordance to achalasia based on BS studies and HRM studies. Prevalence written in the quantitative format "number of achalasia patients fulfilling MIIT threshold (percentile from total achalasia found)/number of non-achalasia patients fulfilling the MIIT threshold (percentile from total non-achalasia found)". MIIT: multichannel intraoesophageal impedance transit; HRM: high-resolution manometry; BS: barium swallow

MIIT threshold	BS study	HRM study	χ2	p-value
>3.29 minutes	78 (73.6%)/14 (6.17%)	96 (99%)/52 (6.39%)	11.3	<0.001
>4.05 minutes	77 (72.6%)/11 (4.85%)	96 (99%)/31 (3.80%)	4.69	0.030
>5.25 minutes	76 (71.7%)/9 (3.96%)	95 (97.9%)/22 (2.70%)	2.56	0.110
>5.45 minutes	76 (71.6%)/8 (3.52%)	94 (96.9%)/22 (2.70%)	3.41	0.065

## Discussion

MIIT is a concept to measure oesophageal transit that is being recommended to utilise during the MII-pH study. In this study, MIIT was able to explain the dysphagia symptoms in patients from the oesophageal transit/retention, and MIIT was also exceptionally reliable to the gold-standard clinical tests used to assess the oesophageal motility and the oesophageal transit. The MIIT test can easily be incorporated into the MII-pH study as a complementary or adjunctive test for transit, and its adoption would permit practitioners to undertake parallel assessments of oesophageal transit and motility for the comprehensive evaluation of the oesophageal physiology. MIIT test has been shown to replicate BS study outcome for achalasia in this study and therefore can cater to the CCv4.0. In light of this, the patient may be exempted from undertaking further testing in radiology and adverse effects affiliated with the BS study (X-ray exposure, consumption of barium sulphite, etc.). Not needing to refer patients for BS study after HRM would eliminate the delay in confirming the diagnosis from transit testing and also the delay for the patient receiving treatment (eliminating the delay would reduce the risk of developing irreversible anatomical abnormality of the oesophagus). Furthermore, MIIT could potentially be the alternative test for BS study when it is contraindicated by some clinical conditions (i.e. pregnant women would be risking birth defects by X-ray exposure, patients with pre-cancerous Barrett's oesophagus, presence of perforation or fistula within the gastrointestinal tract would risk organ leakage of barium sulphite contrast, etc.). The MII-pH and MIIT studies are not contraindicated in these conditions. From the safety of conducting MIIT, it would not pose additional risk or burden to patients than undergoing the MII-pH study as part of their standard clinical care. The clinical benefit of performing the MIIT test (during the MII-pH study) would outweigh the side effects (i.e. dislike for salt). Likewise to BS, MIIT could possibly direct patients' treatment pathways.

BS and HRM findings in light of MIIT cut-off thresholds

Twenty-nine of the 106 achalasia patients (27.4%) diagnosed in BS studies had normal MIIT recording (i.e. <5.45 minutes). The HRM on these cases also excluded achalasia in 25 of the 29 patients (two patients had fragmented peristalsis, 17 patients had ineffective oesophageal motility (IOM), four patients had OGJ outflow obstruction (OGJOO), and two patients had absent contractility). Furthermore, HRM excluded achalasia in 36 of the total 106 achalasia patients (34%) who were diagnosed in BS studies. Conversely, in the cohort of patients with normal BS studies (control group), eight of the 227 patients (3.52%) had MIIT exceeding 5.45 minutes which would be consistent with achalasia. On reviewing the HRM of these eight patients, six of the eight patients did have achalasia, and the remaining two of the eight patients were diagnosed with OGJOO (their OGJOO diagnosis was based on observing remaining segmental peristalsis randomly occurring which raised the suspicion of early stages of achalasia developing).

In the cohort of patients with achalasia diagnosis based on HRM studies, MIIT duration of >5.25 minutes was detected in 95 of the 97 patients (thus, MIIT concordance to HRM for achalasia is 97.9%). Furthermore, in the two cases where MIIT did not fulfil the criteria for achalasia (i.e. MIIT <5.25 minutes), the MIIT findings in these two cases were borderline at 4.2 minutes and 4.7 minutes (both patients had the achalasia diagnosis on BS study). These two cases would have been classed as achalasia as the stronger affinity to achalasia was found from 4.05 minutes when both HRM and BS studies showed concordance for achalasia for high sensitivity (100%) and PPV (94.4%). In the cohort of patients excluded from achalasia on HRM studies, 21 of the 814 patients (2.58%) had MIIT exceeding 5.25 minutes. Of these 21 patients, one patient had IOM with normal BS study, and seven patients had OGJOO (two of the seven patients were diagnosed with OGJOO based on a single peristaltic event and they are suspected of developing achalasia as previously mentioned, and one of the seven patients in this cohort had achalasia diagnosed on BS study). The remaining 13 of the 21 patients had absent contractility diagnoses which were absolute based on standard water swallows and adjunctive testing [9]. Notably, nine of the 13 patients with absent contractility diagnosis additionally showed similar integrated relaxation pressurisation (IRP) to the LOS tone, which raised the suspicion of achalasia [12,13]. The findings here suggest MIIT may detect achalasia in the state of normotensive IRP which is undetected by HRM.

Patients with concordant findings of achalasia on both BS and HRM studies have demonstrated MIIT time to exceed 4.05 minutes. In contrast, where achalasia was excluded from both BS and HRM studies, only four of the 209 patients (1.9%) from this cohort had MIIT >4.05 minutes in the distal oesophagus. Of these four patients, three patients were diagnosed with OGJOO, and one patient was diagnosed with IOM (but the IOM case also revealed the LOS tone to be similar to the IRP of the OGJ which raises the suspicion of OGJOO with normotensive IRP). We have arranged follow-up HRM studies for these four patients in 12-18 months to re-assess the development of achalasia.

Technical principles and feasibility of MIIT

MIIT was possible by the fixed arrangements of the paired impedance sensors within the oesophageal body that measure saline transit during the MII-pH study. The segregated paired impedance sensors act as local "checkpoints" at fixed locations along the oesophageal body which measure the passing flow of ionic charges carried by saline. The SBI (0.9% w/v NaCl) was found to have a nadir impedance of <200 Ω (mean and median) in the laboratory. Furthermore, this threshold of SBI was observed after soaking the MII-pH catheter in saline for 10 minutes under laboratory conditions. The initial SBI was as high as 340 Ω (see Figure 1, impedance sensor z5). The approximated nadir impedance of saline (or SBI) was observable during MIIT and found to be distinguishable from the MBI. MIIT recordings can be identifiable at multiple impedance range settings (from 500 Ω to 4000 Ω) because of the significant distinction between SBI and MBI.

Our findings of the MBI in patients found in the control group (patients excluded from achalasia) were similar to basal mucosal impedance reported in patients with healthy oesophageal mucosa [14]. Incidentally, we observed the MBI in patients from the case group (achalasia) to be reduced (95% CI: 634.6-1078 Ω) and closely resembling the threshold found in patients with reflux oesophagitis [14]. The clinical significance of low MBI findings pertains to gastroesophageal reflux disease which was unexpected in our cohort of patients with achalasia because they had no or very little oesophageal acid exposure. The reduced MBI in achalasia patients may be from the presence of residual bolus in the oesophagus throughout the nocturnal period and not from mucosal acid injury. Other researchers have also reported reduced MBI in patients with achalasia [15]. The findings of low MBI should therefore be taken with caution to predict impaired mucosal integrity or acid mucosal injury. Moreover, the MBI threshold may need to be re-assessed in patients with normal and inflamed oesophagus with respect to the oesophageal motility disorder.

Transit measurements in low levels of oesophageal impedance may be difficult to interpret which was observed in the previous studies using HRMZ [6,16]. This issue may be explained by the nadir impedance of the barium sulphite being closely resembling the MBI in achalasia patients. The nadir impedance of barium sulphite is not known in the literature, and the investigators do not report conducting laboratory tests to determine the baseline impedance of barium contrast. Simply, there may be no means of interpreting the barium sulphite transit on impedance sensors in achalasia patients. This problem was not encountered during our MIIT technique using saline which underwent laboratory tests for the SBI, and the SBI was revealed to be significantly lower than MBI even in achalasia patients. Thereby, the saline transit was identifiable from entering and exiting the oesophageal lumen in achalasia and non-achalasia patients (see Figure 2).

Other researchers have used mixed barium contrast with saline as a single transit marker [3,17]. Theoretically, salt concentration would be diluted in the saline by the mixture and may increase in the SBI signal that could potentially be closer to the MBI (especially in patients with achalasia). This could still make the saline transit within the oesophageal lumen difficult to interpret in patients with achalasia. Secondly, the use of barium contrast for transit measurements poses additional problems, such as the viscosity of the new mixture may still leave residue smeared on the impedance sensors after entering the stomach [3,6]. This may cause confusion in interpreting the bolus retention from oesophageal clearance. In our method, we found the SBI of uncontaminated saline to be significantly lower compared to the oesophageal MBI in patients from the control group (achalasia excluded) and from patients in the case group overall (achalasia patients). A detailed analysis of the technical readings of MBI in the case group revealed that 14 of the 64 patients (21.9%) had MBI less than 200 Ω owing to residual bolus stasis throughout the night. The interpretation of saline transit in these 14 patients may only pose difficulty to an inexperienced practitioner to identify swallows (or antegrade flow) on MII-pH recording. As MIIT is a new concept being introduced to measure oesophageal transit, currently there is no assistive tool/software or auto-scanning function to detect MIIT studies.

HRMZ is not routinely used for oesophageal transit studies and is currently used for research purposes. To date, there seems to be no standardisation in the method or protocol to perform transit studies on HRMZ study which also leaves MIIT to adopt a variation in protocols. The literature has documented a variation with regard to (i) the quantity of fluid volume used for conducting the oesophageal transit test, (ii) the type of transit substance used (solid, semi-solid and liquid) and (iii) the swallowing method adopted (rapid swallowing or standard swallows). The permutation from all three is likely to produce different standard normal ranges for MIIT. Transit studies on HRMZ using rapid swallowing method have been found using 100 ml and 200 ml volumes of barium contrast that revealed dissimilar results [6,16]. On the standard swallow method, researchers have used liquid volumes of 5 ml and 10 ml [3,4,11,15]. Researchers have also soaked bolus products in saline to observe the saline impedance signal with solid swallows [3,15,17]. In our method, we were able to clearly identify the saline transit from using uncontaminated saline, and the rapid swallowing technique seems to be the best method for measuring bolus retention on HRMZ [7]. Our protocol adopted the rapid drinking of 200 ml volume of saline for the MIIT test. However, our transit test differed from the HRMZ study as we were not restricted by the limited study timeframe affiliated with the HRMZ study. Thus, we were able to objectively measure the full duration period of the intraoesophageal saline retention. This permitted calculation for MIIT thresholds for the 5-95% confidence interval for achalasia, and we were able to predict achalasia based on MIIT with respect to the BS and HRM outcome. The MIIT test is not dependent on the HRMZ hardware system (HRMZ catheter and Manoscan module) which is relatively more expensive compared to the MII-pH catheter and the ZepHr® Impedance/pH reflux monitor. Also of note, the HRMZ device is not widely available in many gastroenterology centres and used relatively less than HRM and MII-pH tests. Using an MII-pH device to measure the oesophageal transit has not been reported in the literature to the best of our knowledge.

We noted that all patients excluded from achalasia on HRM were able to successfully drink the 200 ml volume of saline within 20 seconds. This may be because non-achalasia patients do not have bolus retention above the OGJ and this cohort of patients did not report any symptoms afterwards. Therefore, our MIIT protocol for rapidly swallowing 200 ml technique showed some degree of success. The patients with achalasia diagnosis on HRM were mostly able to drink the 200 ml volume of saline but required more time (~30 seconds), and approximately a fifth of them were not able to drink the full 200 ml volume of saline (this may be from oesophageal retention or severe delay in oesophageal clearance to rapidly swallowing saline). We measured the remaining volume of saline, and we can confirm that at least 150 ml of saline was drunk by the achalasia patients. We did not set a requirement for the patients to drink the full 200 ml volume of saline as a caution to prevent regurgitation or vomiting from possible oesophageal retention. However, 200 ml volume was a reasonable volume of fluid for the oesophagus to accommodate in the control group patients which was also used as the standard volume of barium contrast for the BS studies. Conversely, not being able to rapidly drink the 200 ml volume, owing to suspected poor oesophageal transit/clearance, may also raise the suspicion of achalasia. The entry and exit of saline from the oesophagus were easily identifiable during the MIIT test, and the distal oesophageal pH sensor remained stable at neutral pH during the retention of saline (see Figure 2). No change in the distal oesophageal pH from neutral in addition to the absence of retrograde flow capture on the impedance sensors suggests the absence of a reflux event occurring during the MIIT test. None of the patients had regurgitation or vomiting symptoms while in the clinic for 15 minutes after drinking the saline. There were no reports made by patients in their diaries of regurgitating or vomiting the saline.

Researchers have attempted to use food and drink bolus to measure the oesophageal transit during HRMZ studies [2,15]. One study compared the oesophageal transit on standard swallows of liquid and viscous substance (applesauce) in conditions of normal oesophageal motility, simultaneous contractions and absent contractility and found the viscous substance to have a much longer transit time in each motility condition [4]. The findings suggest that both motility and the density of the bolus being swallowed influence the transit time. Therefore, the choice between saline, barium contrast and food bolus (or a mixture bolus) should be carefully considered with respect to known normal transit ranges or before conducting laboratory tests for baseline assessment. One study used an Osmolite formula and yoghurt for the oesophageal transit assessment and reported finding aerophagia (or air entrapment in the oesophagus) that masked the impedance recordings in 38% of their patients [15]. In our protocols, the rapid swallowing of saline during the MIIT test was via sipping and swallowing through a straw as opposed to drinking from a cup. Patients were able to exhale between the swallows during the MIIT test (if necessary). Such techniques were employed for the MIIT test to minimise the risk of aerophagia or swallowing air during the MIIT test. We did, however, still observe air trapping in 12 of the 97 patients (12.4%) who were diagnosed with achalasia on HRM. There was residual air stenosis captured in the proximal and the mid oesophagus in these patients which masked the impedance transit recording of the proximal and mid oesophagus. The MIIT was not interpretable in the proximal and mid oesophagus for these 12 patients, but the distal oesophageal MIIT, where saline resided, was not affected. The distal oesophageal MIIT was interpretable of the saline retention. In light of air trapping in the proximal and mid oesophagus, the distal oesophageal MIIT duration may be the most accurate and possibly more clinically relevant to practice which is why MIIT cut-off thresholds were measured from the distal oesophagus. From the perspective of pathological acid reflux, the MIIT of the distal oesophagus would be a relevant indicator for investigating the mechanism of pathological reflux from poor oesophageal acid clearance as opposed to frequent reflux events occurring. From the perspective of oesophageal motility, the distal contractile integral (DCI) on HRM is the contractile matrix index for measuring the peristaltic vigour and motility which will be associated with the effective clearance of the distal oesophagus. The DCI has not been studied alongside the oesophageal transit on HRMZ, and we have not studied the DCI in correlation to distal oesophageal MIIT.

We also did observe the intraoesophageal transmission of air during the MIIT test in patients who were in the control group (i.e. excluded from achalasia). Interestingly, the recording of air entering the oesophagus was observable only in the final stage of swallowing saline and was represented by a single impedance waveform of air (>10,000 Ω) which was trekking from the proximal oesophagus to the distal oesophagus (there was no stagnation of air in the oesophagus as seen in the 12.4% of achalasia patients). The exiting of air from the oesophagus also revealed clearance of saline and impedance recovery to the pre-saline MBI level. This could suggest that the transmission of air from swallowing in the mouth and travelling into the oesophagus and stomach without stagnation in the passage is a normal phenomenon. The stagnation of air by entrapment in the proximal and mid oesophagus in achalasia patients was due to stasis of saline in the distal oesophagus (or above the OGJ). The saline retention in the distal oesophagus was owing to the non-relaxation of the LOS. The detection of air entering the oesophagus on swallows during the transit studies may uncover techniques to diagnose aerophagia from the MIIT tests.

Technical comparison of MIIT HRMZ and BS

The pitfalls of transit study on HRMZ have been reported [6] which are not found in MIIT during the MII-pH study. In a nutshell, MIIT on the MII-pH study does not have incoming manometry waveforms that confound the impedance readings of transit (as observed on HRMZ), nor does the MIIT technique on MII-pH produce a subjective interpretation of oesophageal retention based on purple colour gauge like HRMZ (MIIT reading of impedance is a simple objective graphical representation). In terms of the physical dimension of the catheters, the HRMZ catheter has a much larger cross-sectional diameter (12.6 Fr) compared to the MII-pH catheter (6.9 Fr) and therefore will occupy a larger cross-sectional area along the oesophagus and opening of the OGJ. The MII-pH catheter (used for the MIIT test) is more cost-efficient and seems more feasible to acquire compared to the HRMZ catheter (the MII-pH catheter is ≈£100 per unit, while the HRMZ catheter is ≈£12,000 per unit).

Based on the data obtained in this study, MIIT has demonstrated greater sensitivity for detecting achalasia than the BS study and a statistically higher degree of concordance to the HRM study which is currently the gold-standard test for achalasia. The MIIT test does not expose the patient to radiation and can be performed on patients who are contraindicated for BS study with X-ray imaging. The ultimate test for MIIT is being able to detect varying transit rates in other non-achalasia motility disorders which the BS study could not detect as abnormality (in this study, they were mostly found as normal). Despite HRM being the gold standard for achalasia, MIIT has demonstrated detecting achalasia with normotensive IRP which was further confirmed by a BS study. MIIT may be detecting early stages of achalasia in patients who are showing diminishing peristalsis on HRM which is a unique feature that other modern-day tests cannot capture. We will conduct a follow-up HRM and BS study in 12-18 months to assess the development of achalasia.

Limitations of the MIIT concept and this study

As MIIT is being introduced and tested in this study, we discovered limitations in the MIIT concept and technique which should be discussed for writing standard operating protocols to perform MIIT in general or conducting further clinical research. Firstly, we noted that not all patients could drink the full 200 ml of saline and certainly not at the same rate. To address the volume, we can confirm all patients did drink at least 150 ml saline from both the case group and control group, and in the literature, other studies have documented using 100 ml volume drink [16]. Based on our experience from the current study, 100 ml volume is likely to result in changes to the MIIT finding for the control group but not for the case group (achalasia patients) because MIIT at 200 ml of saline was significantly prolonged in achalasia patients such that drinking 100 ml of saline is likely to still exceed 4.05 minutes. The drawback of conducting MIIT with 100 ml fluid volume may be that patients with dilated distal oesophagus will have saline retention in the distal oesophagus and residual air in the mid and proximal oesophagus. In the absence of a dilated oesophagus in achalasia patients, it is possible that these patients could only manage to drink 150 ml volume of saline because of the complete stenosis or very severe delay in oesophageal clearance from an aggressive form of achalasia (some achalasia patients required 30 seconds to drink the 200 ml of saline but successfully completed the 200 ml). In the control group, we did observe some patients being able to drink the 200 ml volume remarkably quickly (i.e. within 10 seconds) whilst all patients in the control group drank the 200 ml of saline within 20 seconds. In this study, our calculation of MIIT started from the point of drinking the saline, but based on practical experience and observation of the saline drinking rate in patients, it would be more accurate to commence the MIIT calculation from the point of finish swallowing the saline (i.e. end of the swallows). Choosing the point to calculate MIIT from the end of the swallowing saline and not the onset of swallowing is likely to make a difference to the control group patients' MIIT but less likely to the case group patients' MIIT based on the data collected in this study. During the MIIT, there was no gastric acid reflux based on the MII-pH routine analysis of gastric reflux; however, we cannot guarantee if there was a regurgitation of saline ("saline pocketing") or non-acid reflux occurring during the retention phase because low impedance was observed and pH of saline remains at neutral.

Our technical protocol for MIIT did not include standard 5 ml or 10 ml saline swallows which may be a more controlled method to measure the MIIT in the case group and control group patients. However, we first find this to be impractical because the MIIT real-time test is not viewable on the reflux monitor (the MII-pH study file inclusive of the MIIT test was transferred to a PC upon completion of the 24-hour recording to be viewed on the BioView Analysis program). Therefore, we would not be able to supervise or control the number of standard swallows performed by patients during the standard swallow assessment nor observe for sufficient time lapse between swallows. Secondly, the measurement of standard swallows of saline is less applicable to measuring oesophageal transit or retention but more for measuring the rate of reflux clearance or transit velocities. The rapid swallowing of 200 ml of saline conducted in this study permits measuring retention and delay in the oesophageal clearance which was compatible with the BS study protocols (patients were given 200 ml of barium contrast during X-ray imaging for the BS study). There were some drawbacks to comparing MIIT to the standard BS study. Firstly, our patients did not do a timed BS study which would have been more clinically relevant to the MIIT test as time is measured. The second drawback is the barium contrast itself, which is more viscous compared to MIIT, and viscosity influences transit [4]. However, cross-matching timed BS study to MIIT more likely offers more accuracy in the achalasia sensitivity (or detection) than using the standard BS study. It would be interesting to see if the concordance of MIIT to timed barium swallow supersedes the concordance to HRM.

Despite making much effort to minimise the risks of air swallowing (aerophagia) during the MIIT test because it could mask the transit of saline or interfere with the MIIT recording, we did still observe this in both achalasia and non-achalasia patients. MIIT calculation could not be performed at the level of the oesophagus where stagnation of air is observed. We wondered if these achalasia patients were a sub-cohort with a dilated oesophagus and whether they could have drunk more saline to observe for the retention to reach the mid and proximal extent of the oesophagus. As aerophagia transmission of air in non-achalasia patients was also evident, we could consider these patients to undertake biofeedback therapy sessions when suspecting aerophagia from the clinical history of atypical reflux symptoms such as abdominal bloating/distension and pain, belching, etc. occurring predominantly in the post-prandial phase.

Although SBI was significantly different from MBI that permitted the saline transit to be identified during the MIIT, the exact SBI, however, that was found in the laboratory (<200 Ω) is unlikely to be seen in patients on reviewing the MIIT study for a number of reasons. Unlike the laboratory condition, saline is diluted throughout the human testing process: from the oral cavity with mixing of saliva and food particles prior to reaching the oesophagus. In the oesophagus, there may be mucous, residual fluid from regurgitation or gastric reflux which would also dilute the salt contraction of saline. Our laboratory test for SBI was conducted at room temperature without this by-product contamination, whereas the human body temperature is different, solubility changes with temperature, and there are by-products contaminating the saline. Furthermore, under the laboratory conditions, the nadir impedance of SBI was identified actually after 10 minutes of soaking the impedance sensors and not instantly at room temperature and pressure (i.e. upon insertion of the MII-pH catheter into saline, the SBI could be as high as 340 ohms (see Figure 1)). The time factor plays a role such that SBI stabilisation could be seen in achalasia patients where retention is ≥10 minutes. However, the MIIT recording would show an approximation to the SBI found in the laboratory in patients with faster oesophageal clearance, such as the control group patients who have MIIT less than four minutes (95% CI) or 3.3 minutes in asymptomatic patients where nadir impedance stabilisation period is not met intraoesophageally. Taking into consideration all the practical and technical issues, the laboratory SBI can only be used as an approximation for the MIIT to identify the saline transit.

The baseline oesophageal impedance in achalasia patients is found to be reduced in this study and has been reported by another study [15]. This is supposed by the residual bolus particles remaining in the oesophagus. The implication of this in the current study is that MBI being reduced would mean seeing smaller changes in impedance during the saline drinking which may be difficult to observe for the saline retention period and the MIIT time calculations may be lower true values of saline clearance as the expected MBI to recovery is already reduced. Much of the impact of this would be on the clearance rate or MIIT velocity of saline which the current study did not investigate. There are no supporting computerised tools or functions for MIIT measurements of time and transit velocity matrix which would be very useful in cases with reduced MBI such as this. As MBI in achalasia patients is already relatively low and they tend to have prolonged MIIT in this study, eating or drinking immediately after the MIIT assessment would dilute the saline fluid intraoesophageally, and MIIT recording would be falsely perceived as saline transit or oesophageal clearance. There are no protocols defining when patients can resume their eating/drinking after the MIIT test. The protocol should also contain pre-assessing patients for avoiding salt who may be contraindicated from having MIIT (i.e. those patients with hypertension, cardiac conditions, chronic kidney disease, etc.). The contraindicator for MIIT may be different from the MII-pH study.

The MII-pH catheter is designed for measuring gastric reflux and optimising the diagnostic yield and accuracy by measuring retrograde impedance flow of reflux in the oesophageal regions. Thus, the impedance sensor arrangement is situated for measuring reflux, and the impedance sensor location may not be ideal for measuring oesophageal transit. This is based on the MII-pH catheter design (reference ZAI-BG-44) which cannot measure MIIT at the OGJ level, and therefore, the total transit time could not be measured. This catheter design also could not measure MIIT at 1 cm above the OGJ and 2 cm above the OGJ which would be more clinically relevant and compatible to compare to the timed BS study used in clinical practice. Nonetheless, this MII-pH catheter did successfully demonstrate the MIIT concept to measure oesophageal transit and has proven reliable to the standard clinical testing available. If MIIT would be routinely considered as part of the MII-pH investigation, we would advocate the manufacturer making slight modifications to the impedance sensor location that would be applicable for measuring both gastric reflux and oesophageal transit in the proximal, mid and distal oesophagus. This would be to re-design the sensor location at 0 cm, 1 cm, 2 cm, 6 cm, 8 cm, 12 cm and 17 cm above the OGJ.

Although MIIT did not impose additional risk or burden on patients than undergoing their MII-pH study, we did find that not all patients were fully compliant with the MIIT protocol which affected their performance and, in turn, the data captured. A marginal cohort of patients opted not to have the MIIT test when offered to them because of their dislike of salty water (≈2%). The majority of patients oversaw the salty taste and showed interest in the clinical benefits of MIIT which could potentially explain their symptoms. It was notable that patients volunteering to have the MIIT test would attempt to give us feedback on the salty taste of saline after taking a few sips within the MIIT test. We encouraged participants not to talk during the saline drinking and to complete the saline drinking within 20 seconds. We found some patients' dislike for salt was confessed prior to starting the MIIT test and some requested to drink the saline from the cup directly and not through the straw. We found these patients to drink from the cup in two or three big gulps and within 10 seconds. Firstly, to address the taste of saline, manufacturers may aim to tackle this by flavouring the saline to make it more palatable to conduct MIIT. The flavouring should maintain the salt concentration and the neutral pH. Secondly, as MIIT was measured from the onset of drinking saline, the patients' performance in conducting MIIT may impact the findings particularly in patients from the control group because the normal threshold in MIIT was narrow. Here, it may be worthwhile to consider MIIT calculations after the swallowing phase as previously discussed. Furthermore, in this study, our control group patients had other non-achalasia motility disorders like OGJOO (12.8%), absent contractility (13.7%) and IOM (48.2%) which may also influence the MIIT findings, and variation in MIIT in these conditions is beyond the scope of this study. None of the control group participants were healthy volunteers for us to release normal range values of MIIT. The MII-pH study is a minimally invasive test, and recruitment for MII-pH and MIIT may not be easily feasible. In light of this, the recruitment process for participants to undertake MIIT was aimed towards patients who were clinically referred for the MII-pH study as part of their standard diagnostic assessment for dysphagia and reflux symptoms.

The MIIT findings of oesophageal transit were based on uncontaminated saline transit, and we did not mix the saline with barium contrast or solid bolus which would require extending our laboratory testing to determine the SBI of the mixture. Nevertheless, we did observe normal MIIT findings (i.e. <3.29 minutes) in 278 of the 334 patients (83.2%) with positive HODQ scores for clinical dysphagia, and we wondered if the clinical dysphagia could have been explained by solid bolus swallows with the saline mix. If this MIIT study were repeated, the laboratory test would need to extend into detecting impedance of the saline (SBI) in the bolus volume mix solids, along with mix substance density and titration of the mix. The swallows of the new mixture will require a single bolus swallow assessment as rapid swallowing of solids is likely to cause choking. It should be noted that single bolus swallows on the MII-pH study may not be feasible as previously described because MIIT is reviewed upon the completion of the MIIT test. Therefore, patients completing isolated single swallows of the mixture cannot be observed in real-time, and supervision of swallowing cannot be achieved.

In this study, we did observe low MBI in 21.9% of the achalasia patients which we suspected was caused by residual bolus in the oesophagus throughout the night. By the very nature of the achalasia condition, these patients are likely to have residual food particulates in the oesophagus which gives rise to the reduced MBI observed. This is a shortcoming in this study for the case group. Another shortcoming is that the MBI calculation is derived from the MNBI which is only calculated from the nocturnal period with minimal swallows in an unconscious state, whereas MIIT is performed in a conscious state with rapid swallowing of saline.

With regard to the practitioners of the clinical tests, the MIIT test was performed by a single practitioner who conducted the HRM and the MII-pH studies. The BS studies were performed in a separate department and a separate team of radiologists at different grades (specialist registrar trainees and consultants). As the BS study was not performed in all the patients in this study, we wondered if the concordance of MIIT to HRM being greater was owing to the match sampling (i.e. all patients had HRM whereas only 36.6% of patients had the BS study).

## Conclusions

The findings of this study revealed the MIIT concept was successful in explaining patients' clinical dysphagia and may potentially be used to diagnose achalasia. The MIIT test results showed a good correlation to the traditional standard clinical tests that measure oesophageal motility and transit, and the MIIT method may be a suitable alternative testing method where applicable. The interpretation of MIIT recording is simple and objective and can cater to CCv4.0 without requiring secondary BS studies. The MIIT technique does not pose additional risk or burden to patients than having the MII-pH study, and the clinical benefit of performing MIIT outweighs the side effects.

This study was the first of its kind to introduce the MIIT concept in achalasia and non-achalasia patients which was successfully demonstrated in the two extremes. In the process, we observed the MIIT test to have a strong correlation to HRM, and future potential research topics may investigate the dysphagia severity and transit in a wider spectrum of oesophageal motility disorders, such as the subtypes of achalasia, OGJOO, Jackhammer oesophagus, absent contractility and IOM. Many of these conditions cannot be detected in the BS study for which MIIT may capture the variation of transit in each motility disorder.
